# Endotoxaemia in Haemodialysis: A Novel Factor in Erythropoetin Resistance?

**DOI:** 10.1371/journal.pone.0040209

**Published:** 2012-06-29

**Authors:** Laura E. A. Harrison, James O. Burton, Cheuk-Chun Szeto, Philip K. T. Li, Christopher W. McIntyre

**Affiliations:** 1 Department of Renal Medicine, Royal Derby Hospital, Derby, United Kingdom; 2 Department of Medicine and Therapeutics, Chinese University of Hong Kong, Hong Kong, China; 3 School of Graduate Entry Medicine and Health, University of Nottingham, Derby, United Kingdom; Université de Montréal, Canada

## Abstract

**Background/Objectives:**

Translocated endotoxin derived from intestinal bacteria is a driver of systemic inflammation and oxidative stress. Severe endotoxaemia is an underappreciated, but characteristic finding in haemodialysis (HD) patients, and appears to be driven by acute repetitive dialysis induced circulatory stress. Resistance to erythropoietin (EPO) has been identified as a predictor of mortality risk, and associated with inflammation and malnutrition. This study aims to explore the potential link between previously unrecognised endotoxaemia and EPO Resistance Index (ERI) in HD patients.

**Methodology/Principal Findings:**

50 established HD patients were studied at a routine dialysis session. Data collection included weight, BMI, ultrafiltration volume, weekly EPO dose, and blood sampling pre and post HD. ERI was calculated as ratio of total weekly EPO dose to body weight (U/kg) to haemoglobin level (g/dL). Mean haemoglobin (Hb) was 11.3±1.3 g/dL with a median EPO dose of 10,000 [IQR 7,500–20,000] u/wk and ERI of 13.7 [IQR 6.9–23.3] ((U/Kg)/(g/dL)). Mean pre-HD serum ET levels were significantly elevated at 0.69±0.30 EU/ml. Natural logarithm (Ln) of ERI correlated to predialysis ET levels (r = 0.324, p = 0.03) with a trend towards association with hsCRP (r = 0.280, p = 0.07). Ln ERI correlated with ultrafiltration volume, a driver of circulatory stress (r = 0.295, p = 0.046), previously identified to be associated with increased intradialytic endotoxin translocation. Both serum ET and ultrafiltration volume corrected for body weight were independently associated with Ln ERI in multivariable analysis.

**Conclusions:**

This study suggests that endotoxaemia is a significant factor in setting levels of EPO requirement. It raises the possibility that elevated EPO doses may in part merely be identifying patients subjected to significant circulatory stress and suffering the myriad of negative biological consequences arising from sustained systemic exposure to endotoxin.

## Introduction

Anaemia commonly occurs in patients with chronic kidney disease (CKD), as a result of insufficient production of erythropoietin (EPO) by the kidneys. Effective treatment of anaemia has been possible since the introduction of recombinant Human Erythropoetin therapy in 1986 [1].

Initial observational studies of EPO in CKD suggested a reduced risk of mortality with increasing haemoglobin levels, associated with improved quality of life [2], [3], [4]. However, several clinical trials in recent years have raised significant concerns regarding the optimum haemoglobin targets and erythropoietin stimulating agent (ESA) doses [5], [6], [7]. The randomised trials of Normal Haematocrit Cardiac Trial [8] and TREAT, comparing lower to higher haemoglobin (Hb) targets on composite endpoints, have demonstrated an increased risk of cardiovascular (CV) events. These include stroke and vascular access thrombosis and a potential increased risk of death and provide no clear signal concerning improved patient quality of life.

There remains controversy over whether the poorer outcomes are due to higher absolute levels of haemoglobin or elevated EPO dose, particularly in those patients who fail to achieve target haemoglobin [9]. Patients who do not achieve Hb targets despite elevated doses of EPO, or who require higher doses to maintain their Hb are considered to be Erythropoietin Resistant or Hyporesponsive [10], [11].

Resistance to EPO, previously identified as a predictor of mortality risk [12], is widespread. Iron deficiency is recognised as a significant factor affecting ESA response, and is usually treated alongside erythropoietin replacement as part of co-ordinated anaemia management in the CKD population. Other important factors modulating the individual’s response and potentially increasing ESA resistance include infection, hyperparathyroidism, inadequate dialysis, malnutrition and chronic inflammation [13], [14].

Despite attempts to identify and treat known risk factors, ESA resistance cannot always be explained, suggesting that there may be alternative causes driving the condition. The drivers of systemic inflammation are often obscure. In order to more fully understand the complex relationship between EPO dose, Hb, clinical condition and the dialysis process itself, we identified circulating endotoxin as a potential factor influencing EPO response.

Endotoxin (without sepsis) was initially proposed as a stimulus for immune activation in the pro-inflammatory state of congestive heart failure (CHF) [15]. Endotoxin is released by bacterial cell wall breakdown, within and beyond the gut lumen, from effective host defence mechanisms and by autolysis. Endotoxin enters the circulation via bacterial translocation, passage of intact bacteria and macro-molecules such as endotoxin across the intestinal barrier [16].

Exposure to endotoxin, a profoundly pro-inflammatory stimulus, results in release of a wide variety of pro-inflammatory cytokines, and has been implicated in a broad range of other pathophysiological responses, including oxidative stress, endothelial dysfunction and impaired circulatory autoregulation [17], [18]. We have recently reported significant incremental endotoxaemia with worsening renal function across the range of CKD, with levels roughly tripling after initiation of dialysis [19]. In HD patients the severity of endotoxaemia was associated with both the drivers (ultrafiltration volume and rate) and the consequences (dialysis induced myocardial injury) of dialysis induced circulatory stress.

We hypothesised that there was a potential link between endotoxaemia and EPO resistance in HD patients, and aimed to describe the relative contribution of circulating endotoxin levels to EPO resistance in HD patients.

## Methods

### Objectives

This study aims to explore the potential link between previously unrecognised endotoxaemia and EPO resistance in HD patients.

### Ethics Statement

Ethical approval for the study was granted by Derbyshire Local Research Ethics Committee. Written informed consent was received from all participants.

### Participants

Fifty prevalent HD patients were recruited from a single hospital-based haemodialysis unit. All patients were haemodialysed thrice weekly via native arterio-venous fistulae. Exclusion criteria comprised; change in target weight in the preceding six weeks, clinical evidence of blood loss, active infection or malignancy, bone marrow disease or haemoglobinopathy, or pre-existing severe LV systolic dysfunction (NYHA IV).

### Methodology: Description of Procedures or Investigations Undertaken

#### Haemodialysis details

Dialysis was performed using Hospal Integra monitors (Hospal, Mirandola, Italy). Dialysate fluid contained sodium, 138 mmol/L; potassium, 1 mmol/L; calcium 1.25 mmol/L; magnesium, 0.5 mmol/L; bicarbonate, 32 mmol/L; glucose, 5.6 mmol/L; and acetate, 3 mmol/L. Dual pass water treatment was used with undetectable levels of endotoxin throughout study duration.

All studies were conducted after a 2 day interdialytic period. Anticoagulation was with unfractionated heparin. Dialysate flow was 500 mL/min, and dialysate temperature was set at 37°C. Net fluid removal was set on an individual basis according to ideal dry weight. Patients were permitted to eat during HD if this was their usual preference.

#### Ultrafiltration rate

The rate of volume removal at dialysis, expressed in ml/h/kg BW, measured by the weight change per duration of HD treatment using the post HD weight as denominator.

#### Data collection

The following basic demographic information was obtained: age (years), sex, dialytic vintage (months), cardiovascular comorbidities, diabetes mellitus, body weight (BW; kg), body mass index (BMI; kg/m^2^). The following factors were collected during the dialysis session: interdialytic weight gain (IDWG, kg), ultrafiltration volume (l), ultrafiltration rate (UFR: ml/h/kg body weight), pre-HD systolic and diastolic blood pressure, mean arterial blood pressure (MAP; mmHg), dialysis dose (Kt/V).

#### Erythropoietin dose

In order to normalize the amount of EPO required depending on the severity of anaemia, we calculated an EPO resistance (responsiveness) index (ERI), as described in previous studies [20], defined as the weekly EPO dose divided by Hb level (g/dl). Both the EPO dose and ERI were divided by target body weight to indicate the required EPO dose per kilogram of dry body weight.

#### Blood samples

All blood samples were taken before and after a dialysis session with rapid separation of serum and storage at −85°C before endotoxin measurement. Patients were not fasted prior to blood sampling. Haemoglobin (Hb), ferritin, reticulocytes, serum sodium, potassium, urea, creatinine, albumin, corrected calcium, albumin, and intact parathyroid hormone (PTH) were analyzed using standard autoanalyzer techniques (Roche diagnostics modular IIP®). Commercially available enzyme-linked immunosorbent assay (ELISA) kits (DRG instruments, Germany) were used to assess high-sensitivity C-reactive protein (hsCRP) and Interleukin-6 (IL-6), according to the manufacturer’s protocol.

#### Circulating endotoxin level measurement

The method of lipopolysaccharide (LPS) quantification has been described previously [21]. Briefly, serum samples were diluted to 20% with endotoxin-free water and then heated to 70°C for 10 minutes to inactivate plasma proteins. Serum LPS was then quantified with a commercially available Limulus Amebocyte assay (Cambrex, Verviers, Belgium), according to the manufacturer’s protocol. The detection limit of this assay was 0.01 EU/ml. Samples with LPS level below the detection limit were taken as 0.01 EU/ml. All samples were run in duplicate and background subtracted.

#### Statistical analysis

Results are presented as mean ± standard deviation (SD) or the median and interquartile range (IQR) unless otherwise stated. All data were tested for normality. Categorical data were compared using Chi-square test, continuous data using paired or unpaired Students t-test or one-way ANOVA with Tukey’s correction as appropriate. Correlation between continuous variables was examined by Pearson’s or Spearman’s rank correlation coefficient. Factors associated with ERI/circulating endotoxin levels were further explored by a multivariable linear regression model. Analysis was performed using SPSS v16.0 (SPSS Inc, Chicago, IL). P value of less than 0.05 was considered significant. All probabilities were two-tailed.

## Results

The patient characteristics and blood results are summarized in **Table 1**. Mean Hb was 11.3±1.3 g/dL with a median weekly EPO dose 169 IU/wk/kg [IQR 85–257]. EPO Resistance Index for the whole population was 13.7 IU/kg/wk/gm per dl [IQR 6.9–23.9]. Mean pre-HD serum endotoxin levels were appreciably elevated at 0.69±0.30 EU/ml, significantly higher than those of non-CKD patients (0.04±0.01 EU/ml, p<0.001) [22].

**Table 1:** Patient demographics, clinical characteristics and laboratory parameter results.

Parameter	Results
Age (yrs)	62.2±14.7
Gender (Male : Female)	36∶14
Dialysis vintage (months; median [IQR])	38 [18,70]
Ethnicity (%)	
Caucasian	94
Asian	6
Cause of end-stage renal disease (%)	
Diabetic nephropathy	28
Glomerular disease	22
Adult polycystic kidney disease	12
Urological	10
Multiple myeloma	4
Tubulointerstitial nephritis	4
Unknown	10
Other	10
Diabetes Mellitus (%)	38
Cardiovascular Comorbidities (%)	42
EPO dose (IU/week)	10,000 [7,500–20,000]
ERI (IU/kg/wk/g/dl)	13.7 [6.9–23.9]
Weight (kg)	78.9±17
Body Mass Index (kg/m^2^)	27±5.5
Kt/V _urea_	1.3±0.2
Ultrafiltration volume (Litres)	1.97±0.76
Predialysis systolic BP (mmHg)	144±22
Predialysis diastolic BP (mmHg)	76±14
Haemoglobin (g/dl)	11.3±1.3 g
Haematocrit (%)	36±4
Ferritin (ug/L)	307 [213–454]
Phosphate (mmol/L)	1.45±0.39
Adjusted Calcium (mmol/L)	2.4±0.13
Albumin (g/L)	36±3.8
Parathyroid Hormone (ng/L)	240 [96–342]
hsCRP (mg/L)	1.32 [0.82–2.23]
IL-6 (pg/ml)	0.099 [0.086–0.115]

Data are mean±SD or median [IQR].

ERI, EPO Resistance Index, BP, Blood pressure; hsCRP, high sensitivity C Reactive Protein; IL-6, Interleukin 6.

Predialysis endotoxin levels correlated to both EPO dose and natural logarithm (Ln) of ERI (r = 0.318, p = 0.03 and r = 0.324, p = 0.03 respectively). EPO dose and Ln ERI demonstrated a stronger relationship with ET than with traditional markers of inflammation, including high sensitivity C-Reactive Protein (hsCRP), Interleukin-6 (IL-6) and albumin. Endotoxin demonstrated a trend towards correlation with haemodynamic instability, assessed by maximum drop in systolic blood pressure over the HD treatment (r = −0.270, p = 0.063), as did ultrafiltration volume (r = −0.270, p = 0.058). Endotoxin levels were not significantly affected by the presence of diabetes mellitus (p = 0.61) or aspirin use (p = 0.56).

Ln ERI correlated significantly with ultrafiltration (UF) volume (r = 0.332, p = 0.026), a known driver of circulatory stress previously identified to be associated with increased intradialytic endotoxin translocation. Adjusting UF volume for body weight (L/kg), further strengthened this relationship (r = 0.419, p = 0.004). Ln ERI demonstrated a trend towards correlation with hsCRP (r = 0.281, p = 0.075) and inversely with BMI (r = −0.259, p = 0.082).

Ln ERI was not significantly affected by the presence of cardiovascular comorbidities (p = 0.28), diabetes mellitus (p = 0.78), or RAAS blockade (p = 0.41). EPO dose and Ln ERI did not demonstrate significant correlations with other parameters previously identified as linked to erythropoietin resistance, including ferritin, parathyroid hormone levels, ktV and serum albumin. This patient group were characterised by being well dialysed, iron replete with well controlled hyperparathyroidism. Univariable analysis is summarised in **Table 2**.

**Table 2:** Univariable associates of clinical and laboratory parameters with natural logarithm of EPO Resistance Index.

Parameter	R value	P value
Serum endotoxin (EU/ml)	0.311	0.04
Ultrafiltration volume (L)	0.332	0.026
UF volume/body weight (L/kg)	0.470	0.001
Age (years)	0.057	0.711
Body Mass Index (kg/m^2^)	−0.259	0.082
Ferritin (ug/L)	0.08	0.6
Parathyroid Hormone (ng/L)	0.19	0.25
Kt/V_urea_	−0.18	0.26
Albumin (g/L)	−0.70	0.67
hsCRP (mg/L)	0.280	0.07
IL-6 (ng/ml)	0.16	0.32

UF, ultrafiltration; hsCRP, high sensitivity C Reactive Protein; IL-6, Interleukin 6.

Multivariable analysis of factors contributing to EPO resistance revealed that serum endotoxin and ultrafiltration volume corrected for weight were independent variables associated with the natural logarithm of EPO resistance index in models adjusted for age, albumin, ferritin, PTH and Kt/V (see **Table 3**). In stepwise linear regression, the model predicting ln ERI comprised ultrafiltration volume corrected for body weight (β = 0.472, p = 0.001) and hsCRP (β = 0.301, p = 0.033) with a model fit of R^2^ = 0.297 (Adjusted R^2^ = 0.260).

**Table 3:** Multivariable analysis model for natural logarithm of EPO Resistance Index (adjusted for age, albumin, ferritin, PTH and Kt/V).

	R^2^	Adjusted R^2^	Beta	SE	P value
UF volume/body weight (L/kg)	0.325	0.202	40.3	12.2	0.002
Serum endotoxin (EU/ml)	0.214	0.071	1.06	0.49	0.037

## Discussion

In this study, we demonstrated for the first time endotoxin as an independent determinant of EPO resistance. Significant endotoxaemia has been identified and described in the severe CKD and dialysis population [19], [23] and these data confirm previous findings. Endotoxin levels seen in HD patients are extremely high, comparable with those reported in severe liver disease [24], post gut irradiation [25] and in severe decompensated congestive heart failure (CHF) [15]. Previous work in patients with acute heart failure showed ET to be systemically elevated, with higher levels in the hepatic vein compared to the left ventricle [26], identifying the gut as the source of ET. In CHF, bowel oedema and hypoperfusion have been identified as the two main factors influencing bowel wall permeability [27], and therefore ET translocation.

Factors involved in EPO resistance that can be modulated include iron deficiency, hyperparathyroidism, inadequate dialysis and malnutrition. Chronic inflammation, a common finding in CKD and dialysis patients, is strongly associated with EPO resistance [20]. Elevated circulating pro-inflammatory cytokines (PIC) including hsCRP, IL-6, and TNF-α demonstrate significant correlation with increasing levels of EPO hyporesponsiveness in the dialysis population [20], [28]. A variety of factors have been postulated as drivers of chronic inflammation in CKD, and endotoxin is a potential unifying feature of the interlinked malnutrition, inflammation and CV disease state in dialysis patients.

In this patient group, hsCRP demonstrated a trend towards correlation with ln ERI. This may be attributable to the narrow range of hsCRP values in this patient group, differences in immunoreactivity or insufficient patient numbers to achieve significance. Tachyphylactic response to endotoxin has been previously described, where further contact with, or incremental exposure to endotoxin can result in a diminishing physiological response. The use of native AV fistulae, ultrapure dialysis solution and absence of active intercurrent clinical events such as infection or vascular access issues will affect levels of inflammatory markers in the patient group. The complexities of the uraemic environment, dialysis related and patient specific factors will contribute towards variability in the individual’s inflammatory response.

Exposure to ET results in release of a wide variety of PICs and binding via CD14 to systemic immune competent cells. Mechanisms of cytokine related anaemia include reduction of renal EPO production, inhibition of the proliferation and differentiation of erythroid progenitor cells in the bone marrow, impaired iron absorption and reduced iron delivery [29], [30]. In animal models, induced endotoxaemia has been demonstrated to suppress ESA ability to stimulate erythropoiesis [31].

Endotoxin contamination of dialysis water has long been recognised as a cause of low grade inflammatory response and CV instability during dialysis [32]. Endotoxin exposure in sub-optimally prepared dialysis water has been linked to increased EPO resistance [33], whereas transition to ultrapure dialysate can reduce systemic inflammation and EPO dose requirements [34]. Circulating serum ET levels of 0.69 EU/ml in our patient group were greatly elevated, above even the maximum current permitted levels of endotoxin in dialysate fluid of 0.25 EU/ml. Dialysis water in this study had undetectable levels of endotoxin during the study period, following dual pass water treatment. Circulating ET in these patients therefore originates from an alternate source, namely the gastrointestinal tract.

HD itself appears to be responsible for increasing exposure to translocated intestinal endotoxin, as evidenced by a large difference between patients with very severe CKD stage 5, but not yet started on dialysis, and those receiving dialysis [19]. HD, in combination with ultrafiltration, results in significant systemic haemodynamic perturbation and clinically significant reduction of regional perfusion in critical organs such as the heart and brain [35]. HD is well described as being capable of inducing recurrent cardiac ischaemic injury, associated with long term myocardial damage and increased mortality [36]. Previous work has demonstrated significant correlation between endotoxin and severity of HD-induced cardiac stunning and relative hypotension [19].

Patients on long-term maintenance haemodialysis have evidence of mucosal ischaemia [37] and ultrafiltration causes a reduction in splanchnic blood volume [38] despite preserved blood pressure [39]. Mesenteric ischaemia results in disrupted gut mucosal structure and function, with increased gut permeability [40]. HD may result in recurrent regional hypoperfusion, particularly in the splanchnic vasculature. This can result in subclinical mesenteric ischaemia and injury, leading to altered membrane permeability and increased translocation of endotoxin.

Increasing ERI is associated with higher volumes of fluid removal during dialysis, and unsurprisingly this association increases when UF volume is corrected for body weight. Previous work has demonstrated significant correlation between endotoxaemia and dialysis induced haemodynamic stress, including severity of HD-induced cardiac stunning, markers of cardiac injury and relative hypotension [19]. Ultrafiltration volume is potentially a driver of both myocardial and splanchnic hypoperfusion, with end-organ injury resulting in system specific short-term injury and long-term damage, as well as a generalised inflammatory response.

Adding endotoxin into a simple linear regression model for ln ERI containing UF volume/weight and hsCRP strengthened the R^2^ of the model (0.307), but both ET and hsCRP were no longer independent predictors within it. This is not unexpected, given the underlying pathophysiological processes linking these factors. The relationships between potential causes and consequences of endotoxaemia, may, in turn, influence EPO resistance. Multivariable analysis confirmed the independent association of serum endotoxin and of ultrafiltration volume corrected for weight with the natural logarithm of EPO resistance index, when adjusted for factors previously identified as influencing the response to EPO.

In terms of potential intervention, extended dialysis schedules are associated with marked reductions in UF requirements and intradialytic hypotension, lessening the haemodynamic insult [19], as well as improving Hb and lowering EPO requirements [41].

This study has potential limitations. Although this observational study was able to demonstrate a relationship between endotoxaemia and EPO resistance in HD patients, the sample size is inadequate to fully resolve factors relating to the degree of endotoxaemia or EPO hypo-responsiveness. Patients were not prevented from eating during HD, which could potentially influence gut perfusion and permeability, however the relatively high fibre and low fat meals provided are likely to have only limited impact on ET translocation. Areas of further work include longitudinal studies on ET, inflammatory response and EPO requirements, exploration of the effects of reduced endotoxin exposure on EPO requirements, and comparison of ET and ERI between different dialysis modalities.

### Summary

This study suggests that endotoxaemia, either by direct interaction, or through its well documented effects on systemic inflammation, is a significant and potentially dominant factor in setting levels of EPO requirement. It raises the possibility that elevated EPO doses may in part merely be identifying patients subjected to significant haemodynamic perturbation, and suffering the myriad of negative biological consequences arising from sustained systemic exposure to endotoxin.

A greater understanding of the mechanism and factors influencing endotoxin translocation in the dialysis population is required. In addition, turning our attention to the dialysis procedure itself may yield additional benefits, both in terms of EPO requirements, but also alleviating the haemodynamic impact of HD to improve long-term patient outcomes.
